# Development and Validation of Customized Silicone Shade Guide for Enhanced Aesthetic Integration in Maxillofacial Prostheses: A Cross-Sectional Study

**DOI:** 10.7759/cureus.77893

**Published:** 2025-01-23

**Authors:** Priyadarshani Pawar, Rohit M Patil, Abhishek Y Darekar, Pradnya R Patil, Rutuja B Chavan, Susheel S Chauthmal, Seema Gupta

**Affiliations:** 1 Department of Prosthodontics, Jawahar Medical Foundation's Annasaheb Chudaman Patil Memorial Dental College, Dhule, IND; 2 Department of Prosthodontics, SMBT Institute of Dental Sciences and Research, Nashik, IND; 3 Department of Periodontology, SMBT Institute of Dental Sciences and Research, Nashik, IND; 4 Department of Orthodontics, Kothiwal Dental College and Research Centre, Moradabad, IND

**Keywords:** customized, maxillofacial, prostheses, reliability, shade guide

## Abstract

Introduction: Maxillofacial prosthodontics focuses on restoring facial defects with prostheses, and achieving precise color matching is a significant challenge. Skin pigmentation varies significantly among the Indian population, ranging from light to dark tones, depending on melanin levels. Current shade guides lack the diversity to adequately address this variation. This study aimed to fabricate a silicone shade guide tailored for the Indian population and validate its reliability and clinical utility in color matching.

Materials and methods: This study included 370 Indian participants aged 20-45 years who were selected voluntarily from different regions of India. Skin color was measured using a SkinColorCatch colorimeter (Delfin Technologies, Kuopio, Finland). A 24-shade guide was developed and categorized into light (L1-L8), medium (M1-M8), and dark (D1-D8) tones, using Tech-Sil® S25 silicone (Technovent, South Wales, UK) and intrinsic pigments. Shade tabs with a thickness of 4 mm were used to mimic maxillofacial prostheses. The reliability and validity of the shade guide were tested in 110 participants. Four calibrated observers evaluated the color matching using a shade guide and scored the matches from 1 (poor match) to 4 (perfect match). The inter-observer reliability was analyzed using Cohen's kappa and Fleiss' kappa. Silicone swatches were fabricated for 79 participants with 100% observer agreement, and these swatches were assessed for color-matching satisfaction by the goodness-of-fit test.

Results: The study demonstrated 100% observer agreement for 79 (71.9%) participants, 75% agreement for 27 (24.5%) participants, and 50% agreement for four (3.6%) participants. Cohen's kappa revealed excellent inter-observer reliability, with values ranging from 0.81 to 0.97 among the observer pairs. Fleiss' kappa for all observers indicated substantial agreement (0.85). Silicone swatches constructed for participants with perfect agreement were evaluated for color matching and achieved high satisfaction scores across all observers. Observers reported being "completely satisfied" with the shade guide in 52 (65.8%) to 55 (69.6%) participants, with minimal dissatisfaction. The shade guide demonstrated superior reliability and precision compared with prior studies, highlighting its clinical utility in Indian patients.

Conclusion: This research effectively established a silicone shade guide comprising 24 distinct shades specifically designed for the Indian demographic, organized into light, medium, and dark categories. Each of these categories included eight subshades. The guide exhibited excellent inter-observer reliability, accurate color matching, and substantial satisfaction levels among the observers, thereby providing notable clinical application in the field of maxillofacial prosthetics.

## Introduction

Maxillofacial prosthodontics encompasses the fabrication of artificial substitutes for both intraoral and extraoral anatomical structures, including the eye, nose, maxilla, mandible, esophagus, cranial bones, and palate, with color matching representing a significant challenge [1]. Achieving precise color matching in the human skin is a vital component of the manufacturing process of maxillofacial prostheses [2]. The psychological health of a patient may be adversely affected by nuanced changes in facial characteristics. The application of aesthetic maxillofacial prosthetics can offer support to individuals with diverse difficulties, thereby improving their overall quality of life. Prosthodontists face considerable obstacles in achieving a precise match between the hue of the prostheses and human skin during the fabrication process [3].

Within the heterogeneous global population, individuals of Indian heritage are distinguished by their remarkable spectrum of skin tones, which can be categorized from light to dark, as a result of a multifaceted array of determinants. Although a variety of factors contribute to discrepancies in skin pigmentation, melanin has emerged as the predominant pigment responsible for coloration. Individuals with elevated melanin concentrations generally present with a more profound complexion, whereas those with lighter skin exhibit diminished melanin levels, causing their skin color to be more significantly influenced by the bluish-white connective tissue located beneath the dermis and the presence of hemoglobin within the dermal veins. This complex interaction of biological elements highlights the captivating diversity in skin pigmentation among individuals of Indian ancestry [4]. Consequently, it is imperative to employ tailored shade guides for patients of specific ethnic and racial backgrounds.

Color tabs (4 mm) were integrated into the shade guide to closely mimic the standard thickness of the acrylic resin prostheses. The choice of color must consider this thickness because the underlying anatomical defect may influence the visual outcome of the prostheses. Guttal et al. [5] conducted an investigation to assess the reproducibility of a silicone shade guide in the context of fabricating maxillofacial prostheses for the Indian demographic, evaluating reproducibility through visual assessments conducted by four independent evaluators. The findings indicated that samples representative of darker skin tones exhibited a commendable match with the actual skin tone, demonstrating statistically significant concordance. Nonetheless, most customized silicone shade guides tailored for the Indian population remain unavailable in the commercial market and encompass a limited range of skin tones. Therefore, the primary objective of the present study was to fabricate a customized silicone shade guide for the fabrication of maxillofacial prostheses in the Indian population. The secondary objective was to test the reliability and validity of the shade guide for patients.

## Materials and methods

Study design, setting, and source of data

**Figure 1:**
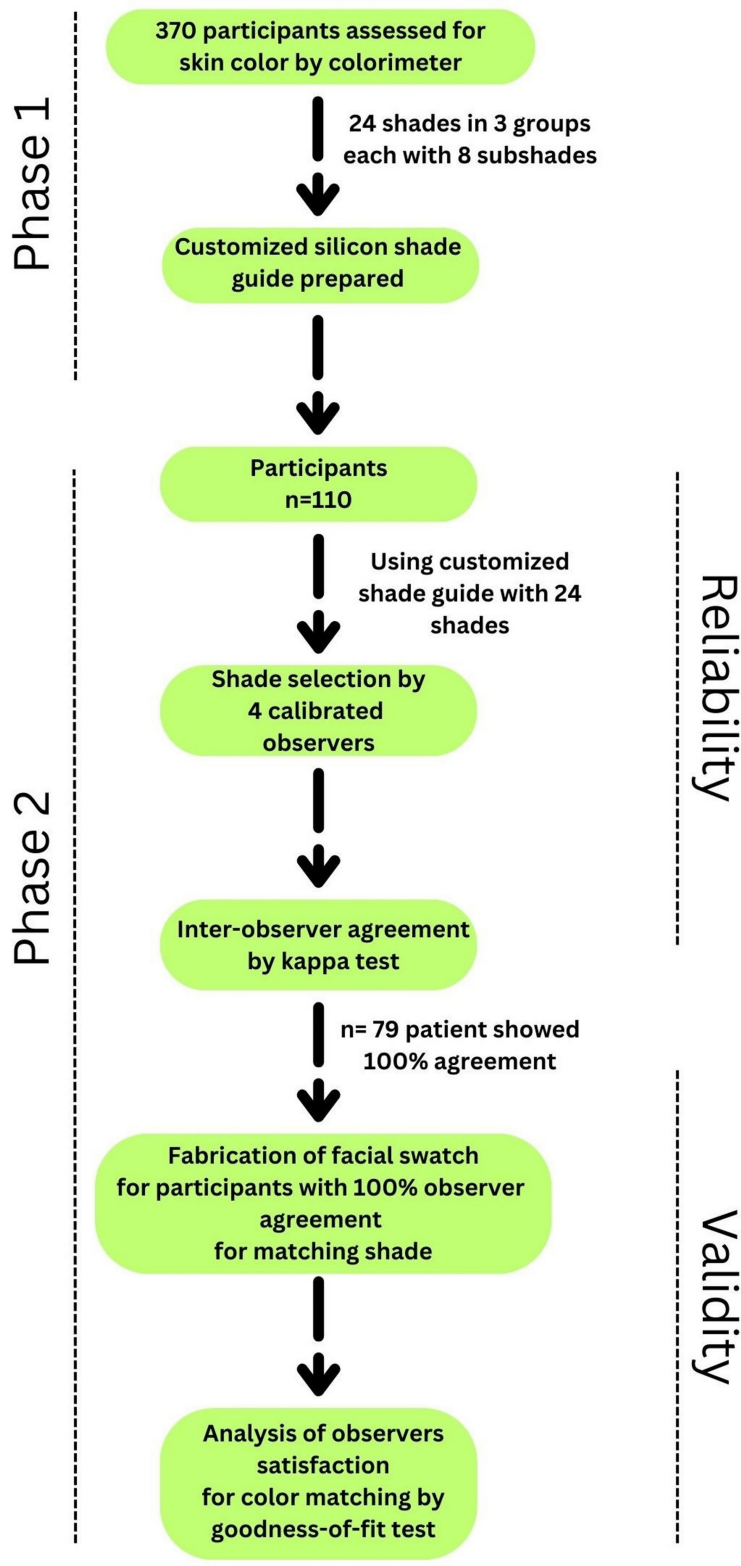
Flowchart depicting the study phases for assessing skin color reliability and validity in participants.

The individuals for this study were selected from various regions of India from the general population on a voluntary basis in the Department of Prosthodontics, Jawahar Medical Foundation's Annasaheb Chudaman Patil Memorial Dental College, Dhule, India, from November 2022 to March 2024. Approval was obtained from the institute's Institutional Ethical Committee (approval number: EC/NEW/INST/2022/2959/2022/003) before starting the study, and the study was conducted in accordance with the principles of the Declaration of Helsinki. All subjects were informed of the study protocol, and written informed consent was obtained. The study followed the Strengthening the Reporting of Observational Studies in Epidemiology (STROBE) guidelines (Figure 1).

Fabrication of customized silicone shade guide

Drawing upon the findings from our prior investigation involving 370 participants mainly from Maharashtra, aged 20-45 years, we utilized a SkinColorCatch colorimeter (Delfin Technologies, Kuopio, Finland), which operates as a visible-spectrum reflectance colorimeter comprising a diode that emits across the entire visible spectrum, to evaluate the skin pigmentation of 370 Indians exhibiting diverse skin shades. The L*a*b* and individual topography angle (ITA) were noted. Consequently, we proposed a shade guide that encompasses 24 distinct colors representing light, medium, and dark skin tones [6], based on these L*a*b* and ITA values.

A customized shade guide was designed based on the proposed shade guide, where pigments were mixed to get the skin color shades as proposed in our previous study, where L*a*b* values after mixing were assessed using SkinColorCatch colorimeter to get almost the same values as obtained in our proposed shade guide. Tech-Sil® S25 silicone (Technovent, South Wales, UK) with a mixing ratio of 10 parts silicone to 1 part catalyst was used as a base material because it is characterized by high-temperature stability, oxidation resistance, high tensile strength, and tear strength and is documented as a stable material over acrylic resin and polyvinyl chloride [7]. The coloring pigments used in the study were sourced from Technovent Ltd., South Wales, United Kingdom, and are listed in Table 1.

**Table 1:** List of materials used in the study with quantity.

S. no.	Code	Product	Unit	Quantity
1	S-25	Tech-Sil 25 Silicone, 10:1	500 g	1
2	P105	Intrinsic master colorant: Brilliant white	15 mL	1
3	P108	Intrinsic master colorant: Brown	15 mL	1
4	P112	Intrinsic master colorant: Brilliant red	15 mL	1
5	P115	Intrinsic master colorant: Brilliant yellow	15 mL	1
6	P405	Grey skin shade	15 mL	1
7	P413	Light buff skin shade	15 mL	1
8	P107	Intrinsic master colorant: Sienna	15 mL	1
9	P111	Intrinsic master colorant: Brilliant blue	15 mL	1
10	P402	Medium brown skin shade	15 mL	1
11	P409	Rose pink skin shade	15 mL	1
12	P419	Mushroom skin shade	15 mL	1
13	P420	Coffee skin shade	15 mL	1
14	P109	Intrinsic master colorant: Black	15 mL	1

The three basic shades were chosen as light (L), medium (M), and dark (D), which were further divided into eight subshades based on the lighter to darker shades in that group. Hence, the L shade was subdivided from L1 to L8, the M shade from M1 to M8, and the D shade from D1 to D8 (a total of 24 shades). The shades and their formulae are presented in Tables 2-4.

**Table 2:** Details of the composition used for light (L) shade in the study. L1-L8: subshades of L shade. All the coloring pigments were added in mL.

S. no.	Code	Subshades of light shade							
		L1	L2	L3	L4	L5	L6	L7	L8
1	P105	0.031	-	0.062	0.093	0.062	0.093	0.031	-
2	P109	-	-	-	-	-	0.015	-	-
3	P405	-	-	0.093	-	-	-	0.062	0.156
4	P111	-	-	-	-	-	-	-	-
5	P115	0.062	0.062	0.125	0.156	0.062	-	0.031	0.062
6	P112	0.062	0.062	0.062	0.031	-	-	-	-
7	P409	-	-	-	-	0.125	0.125	-	0.250
8	P413	0.125	0.125	-	-	-	-	-	-
9	P107	-	-	-	0.031	-	-	-	0.031
10	P419	-	-	0.125	-	0.125	0.125	-	0.125
11	P420	-	-	-	0.093	0.062	-	0.015	-
12	P402	-	-	-	-	0.312	0.375	0.187	0.156
13	P108	-	-	-	-	-	-	-	0.062

**Table 3:** Details of the composition used for the medium (M) shade in the study. M1-M8: subshades of M shade. All the coloring pigments were added in mL.

S. no.	Code	Subshades of medium shade							
		M1	M2	M3	M4	M5	M6	M7	M8
1	P105	0.062	0.093	0.062	0.062	0.250	-	0.031	-
2	P109	0.031	-	-	-	-	-	-	-
3	P405	-	-	-	0.093	-	0.125	0.062	0.250
4	P111	-	-	-	-	-	-	-	0.125
5	P115	0.062	0.093	0.156	0.125	-	0.062	0.093	0.750
6	P112	-	0.093	0.031	0.062	-	-	0.015	0.250
7	P409	0.125	-	-	-	0.062	0.187	-	-
8	P413	-	-	-	-	-	-	-	-
9	P107	-	-	0.031	-	-	-	-	0.125
10	P419	0.125	-	-	0.125	0.250	0.375	-	-
11	P420	0.062	0.093	0.093	-	-	0.125	0.031	0.500
12	P402	0.312	-	-	-	0.250	0.375	0.250	-
13	P108	-	-	-	-	-	-	-	-

**Table 4:** Details of the composition used for the dark (D) shade in the study. D1-D8: subshades of D shade. All the coloring pigments were added in mL.

S. no.	Code	Subshades of dark shade							
		D1	D2	D3	D4	D5	D6	D7	D8
1	P105	0.093	-	-	0.093	0.093	-	-	-
2	P109	-	-	-	0.031	0.062	-	0.062	0.187
3	P405	-	0.250	0.250	-	-	0.125	0.250	-
4	P111	0.031	-	0.031	0.031	0.031	0.062	0.031	-
5	P115	0.125	0.125	0.312	0.375	0.375	0.312	0.437	0.062
6	P112	-	-	0.031	-	0.062	0.125	0.031	-
7	P409	-	-	-	-	-	-	-	-
8	P413	-	-	-	-	-	-	-	-
9	P107	0.187	-	-	0.187	0.187	0.062	-	0.281
10	P419	-	-	-	-	-	-	-	-
11	P420	0.187	0.312	0.500	0.187	0.187	0.250	0.562	-
12	P402	-	0.125	0.125	-	-	-	0.250	-
13	P108	-	-	-	-	-	-	-	-

**Figure 2:**
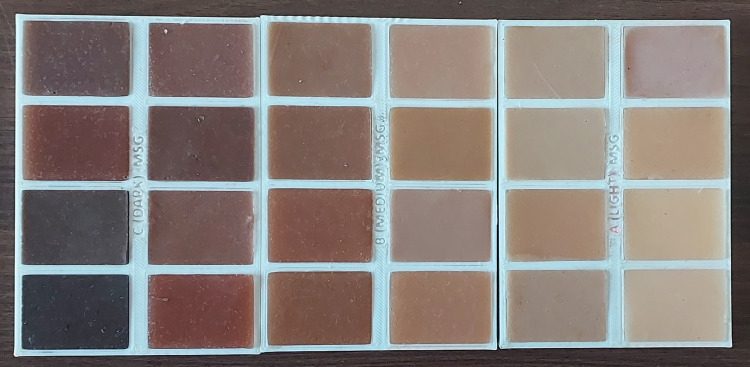
3D printed shade guide consisting of 24 shades, eight subshades of light (L), eight subshades of medium (M), and eight subshades of dark (D). Source: Original work by the author.

A three-dimensional (3D) printed tray for the maxillofacial shade guide was created using 3D printed material from an MK3 Prusa 3D printer (Prusa Research, Prague, Czech Republic) and labeled as A (light), B (medium), and C (dark). Each tray was divided into eight sections measuring 30×25 mm in diameter and 4 mm in depth. Sequential serial numbers (L1-L8 for light shade, M1-M8 for medium shade, and D1-D8 for dark shade) are inscribed on the left corner of each section (Figure 2).

The inner surfaces of the sections were given a matte finish to provide a uniform appearance to the shade tabs. Serrations were incorporated on the outer side of each section, gradually reducing the depth to 0.5 mm. The tray design was specifically tailored to accommodate changes in the thickness of the maxillofacial prosthesis, varying from the middle portion to the peripheral edges. This was done by ensuring that the thickness of the tray decreased towards the outer surface to align with the thickness changes in the prostheses. The different tray thickness layers provide a visual representation of shade variations within the same color palette. Upon loading the silicone material into the designated slot, the shade tab exhibited a matte finish on one side and a glossy finish on the other side after the material sets. This feature allowed for a clear distinction between textural differences when matching the shade with the patient's natural features. With a total of 24 shade tabs, the tray enabled easy and simultaneous shade matching of the patient, streamlining the process for the practitioner. Furthermore, the tray was designed to be lightweight and portable, making it convenient to handle and carry around during the procedure.

The shade tabs were initially fabricated with modeling wax and then invested in dental stone, followed by de-waxing in a conventional manner. Color mixing was performed by mixing approximately 10 g of the base and 1 g of the catalyst, which were precisely measured using a digital scale to ensure accuracy in the fabrication process. The components were then combined in a glass container and stirred with a plastic spatula for two minutes to achieve a uniform and consistent blend. This meticulous mixing procedure is essential for ensuring the quality and integrity of the final product. Subsequently, the intrinsic pigments were meticulously weighed and added to the mixture to create an array of different hues and shades. The silicone compound, enriched with pigments, was carefully poured into the designated base mold, which is a critical step in the production process that requires precision and attention to detail. An insulin syringe was used to label each pigment. Each line in the syringe corresponds to four drops. The incorporation of these pigments not only adds aesthetic value but also allows for the customization and personalization of the final silicone samples, catering to diverse preferences and requirements in the field of maxillofacial prosthetics. This methodical approach to sample fabrication ensures consistency, quality, and versatility in the produced specimens, meeting the standards and expectations of the industry. After pouring the pigmented silicone into the molds, curing was performed for 30 minutes. The processed silicone shade tabs were retrieved from the mold and finished with a fine-grit finishing stone.

Testing the validity and reliability of the shade guide 

The sample size was calculated according to the previous study by Anitha et al. [8], and the kappa agreement of light skin shade in the Indian population was 0.65, medium skin shade 0.32, and dark skin shade 0.27. The sample size for the present study was estimated to be 110 at a confidence interval of 95% and a margin of error of 5% using G*Power Version 3.6.9 (Heinrich-Heine-Universität Düsseldorf, Düsseldorf, Germany).

This study used a convenience sampling approach. One hundred and ten participants of Indian ethnicity who exhibited a similar lifestyle and were devoid of any dermatological issues, including hypo- or hyperpigmentation, burns, facial scars or deformities, skin blanching, and scars or rashes on the upper back and malar area, were eligible, contingent upon their willingness to engage in this non-invasive skin color assessment technique. Individuals with dermatological ailments and those who had undergone tanning, any recorded history of drug-induced reactions, abnormal sun sensitivity, significant sun exposure in the preceding four weeks, skin infections, or inflammation were excluded from the study. All measurements were carried out in a climatized room under controlled ambient conditions (room temperature 22±2°C and relative humidity 45±5%). An acclimatization period of at least 10 minutes was given before the start of every measurement session. The acclimatization period of 10 minutes before assessing skin color was given to ensure that the individual's skin color was measured under stable and consistent conditions. This period allowed the body to adapt to the environmental conditions of the assessment area, minimizing the effects of recent physical activity, temperature changes due to recent exposure to sun, heat, or cold, and other factors that can temporarily alter skin color. The controlled ambient light was provided by the use of light-blocking shades, portable room partitions, and a consistent location for measurements.

**Figure 3:**
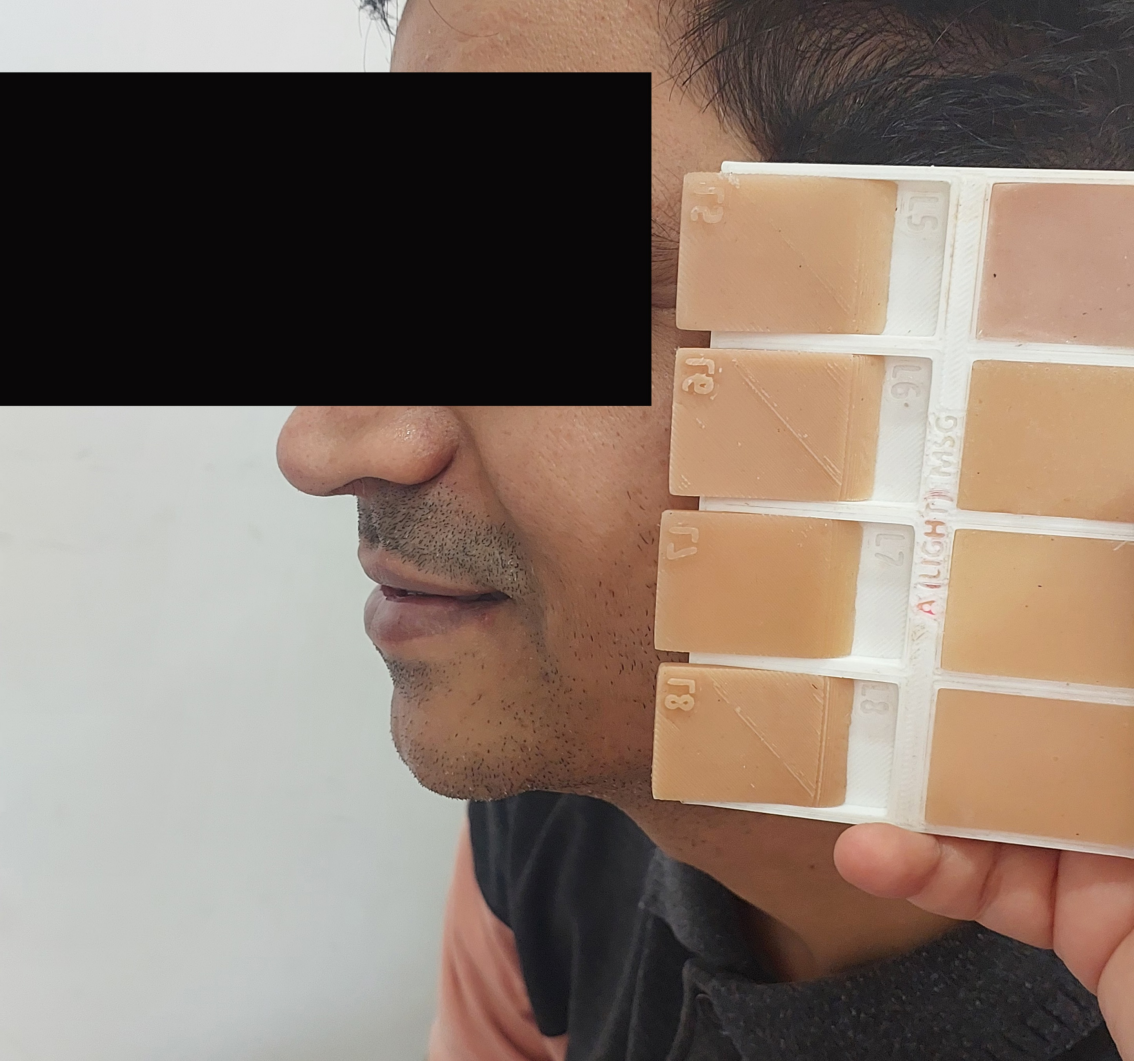
Assessment of the skin shade of the patient using the customized shade guide. Source: This figure represents an image of a patient included in the study, used with consent.

The shade tab was evaluated by four experienced observers (AYD, RBC, SSC, PRP) who were calibrated for the procedure. All observers were briefed on the procedure for skin color assessment using a customized shade guide. Regular practice sessions were conducted in which evaluators assessed shades and received feedback that could help align their judgments. The shade tab was positioned in close proximity to the skin to analyze the level of the match (Figure 3).

The observers were meticulously scrutinized to identify any potential deficiencies in their color vision. To ensure the reliability of their color vision, all observers underwent the Ishihara color test, specifically the Pseudoisochromatic Plate (PIP) Color Vision Test [9], and the results confirmed that they all had normal vision capabilities. Subsequently, each group of observers matched the skin color of an individual with a customized shade guide and proposed shade on the evaluation sheet. To minimize errors, each evaluator made three separate attempts to determine the color match for each subject. Instant identification of the color match upon initial observation was deemed crucial, as the extended duration for matching was restricted to prevent eye fatigue. All four observers were unaware of each other's scores to eliminate bias due to inter-observer variations. Color matching was evaluated through visual assessment using a structured scoring system integrated into a score sheet. The interpretation of the scoring criteria was as follows: A score of 1 indicated a complete lack of correspondence and was deemed inappropriate. A score of 2, as a nearly perfect resemblance, was considered permissible. A score of 3 indicated a satisfactory level of similarity in color and was categorized as acceptable. Precise correspondence in color was acknowledged as correct, with a score of 4.

**Figure 4:**
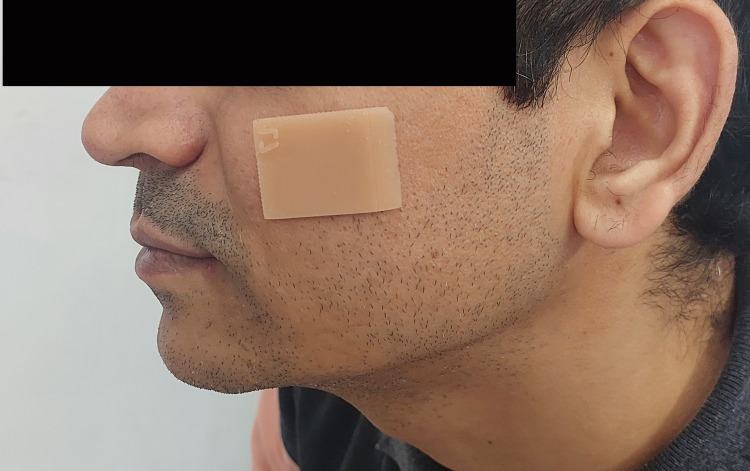
A silicone swatch placed on the skin of the patient for color matching. Source: This figure represents an image of a patient included in the study, used with consent.

In the subsequent phase, silicone swatches were constructed for 79 patients who showed 100% agreement between the observers, and the silicone sample pertaining to each patient was secured to the respective malar area of the dermis by employing a minimal amount of adhesive (Secure Medical Adhesive, Factor II, Wagon Wheel, Arizona, United States), as shown in Figure 4.

The same four observers appraised these swatches for color matching, and the evaluations were performed as previously described.

Statistical analysis

Statistical analysis was performed using IBM SPSS Statistics for Windows, Version 23.0 (Released 2015; IBM Corp., Armonk, New York, United States). The Kolmogorov-Smirnov (KS) test was used to assess the normality of the data distribution. Data distribution with Q-Q plots confirmed normality. To test the reliability and validity of the shade guide, Cohen's kappa coefficient was used to evaluate the agreement among the observers, and Fleiss' kappa was used to assess the reliability of agreement between multiple observers when classifying items into categories. The validity of the shade guide was tested by the chi-squared goodness-of-fit test. Statistical significance was set at p≤0.05.

## Results

**Figure 5:**
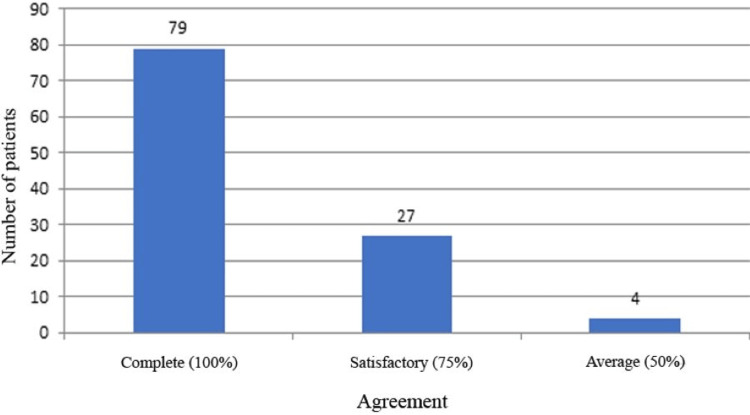
Agreement between observers. The total number of participants was 110, and they were assessed by four observers for the estimation of skin color.

The study demonstrated 100% observer agreement for 79 (71.9%) participants, 75% agreement for 27 (24.5%) participants, and 50% agreement for four (3.6%) participants for skin color assessment (Figure 5).

The inter-observer reliability between different pairs of observers was evaluated using Cohen's kappa coefficient. The highest agreement was observed between observers 1 and 2, with a kappa value of 0.97. The agreement between observers 1 and 3 was slightly lower, with a kappa value of 0.82, followed by observers 2 and 3, which had kappa values of 0.81. Comparisons involving observer 4 showed moderate to substantial agreement, with kappa values ranging from 0.81 to 0.85, demonstrating a high level of consistency in ratings among the different observer pairs, reinforcing the reliability of the assessment method used in the study. The inter-observer reliability among the four observers was assessed using Fleiss' kappa, yielding substantial agreement with a kappa value of 0.85. These results demonstrate a high level of consistency among the observers, supporting the reliability of the ratings in this study (Table 5).

**Table 5:** Inter-observer agreement and reliability analyzed by kappa analysis for the assessment of the skin shade of the participants using the customized shade guide. 0.81-0.99: near-perfect agreement; *p<0.05: significant

Ratings	Cohen's kappa	Standard error	95% confidence interval		Fleiss' kappa	P-value
			Lower	Upper		
Observer 1-observer 2	0.969	0.018	0.935	1.000	0.85	0.001*
Observer 1-observer 3	0.816	0.038	0.741	0.891		
Observer 2-observer 3	0.806	0.039	0.73	0.883		
Observer 1-observer 4	0.846	0.036	0.776	0.917		
Observer 2-observer 4	0.837	0.037	0.764	0.909		
Observer 3-observer 4	0.807	0.039	0.731	0.883		

The results of the chi-squared goodness-of-fit test revealed no significant differences in the distribution of satisfaction levels among the four observers (chi-squared=0.971; p=0.999). The majority of observers rated the shade guide as "completely satisfied" in 52 (65.8%) participants by observer 2 and 55 (69.6%) participants by observer 3. Moderate satisfaction levels were noted in 16 (20.3%) participants by observers 1 and 3 and in 18 (22.7%) participants by observer 2. Mild dissatisfaction ("not satisfied" and "mildly satisfied") was reported at low and comparable rates across all observers. These findings indicated a high degree of agreement among observers regarding their satisfaction levels with the shade guide (Table 6).

**Table 6:** Analysis of the level of satisfaction with the selected shade in participants among the four observers by using the goodness-of-fit test. *p>0.05: non-significant

Level of satisfaction	Observer 1, n (%)	Observer 2, n (%)	Observer 3, n (%)	Observer 4, n (%)	Chi-squared value	P-value
Not satisfied	2 (2.5)	1 (1.3)	2 (2.5)	2 (2.5)	0.971	0.999*
Mildly satisfied	7 (8.8)	8 (10.2)	6 (7.6)	7 (8.8)		
Moderately satisfied	16 (20.3)	18 (22.7)	16 (20.3)	17 (21.5)		
Completely satisfied	54 (68.4)	52 (65.8)	55 (69.6)	53 (67.2)		

## Discussion

In light of the absence of tailored shade guides specifically designed for the Indian population, the current investigation was undertaken to develop a silicone-based customized shade guide comprising 24 distinct shades for the Indian population, as well as to assess its efficacy for clinical application. Empirical research highlighting the advantageous characteristics of silicone elastomers as materials for maxillofacial prostheses has established a basis for the preference of silicone as the predominant material currently advocated [7].

Human skin pigmentation can be evaluated through both subjective and objective assessments, allowing for a comprehensive analysis of this complex trait. In the realm of measuring instruments utilized for this purpose, contact-based devices such as various types of colorimeters and spectrophotometers have frequently been employed in the meticulous fields of maxillofacial prosthetic measurements and extensive skin color research, as documented in sources [10,11]. These measuring devices are designed to make physical contact with the skin's surface, which is essential for capturing and absorbing the totality of reflected light; however, the inherent translucency of the human skin results in a portion of the incident light being scattered away from the instrument's aperture without being absorbed effectively. This phenomenon is referred to as "edge loss," which is recognized as a considerable limitation associated with the functionality of such optical devices [12]. It is important to note that the degree of edge loss correlates directly with both the dimensions of the aperture and beam width within the measuring instrument. Furthermore, measurement inaccuracies can arise from variations in the contact pressure exerted by the measuring devices, which can affect the consistency and reliability of the readings obtained, as indicated in [13]. Consequently, there is a clear need for the development and implementation of a customized intrinsic silicone shade guide, which is specifically tailored for shade selection in clinical practice, thus enhancing the precision and accuracy of color matching for maxillofacial prostheses.

The study's findings underscore a high degree of inter-observer reliability among the observers, as evidenced by both Cohen's kappa and Fleiss' kappa values. This is a critical indicator of the robustness of the customized shade guide used in assessing skin color as it minimizes the variability in subjective interpretations by observers. Anitha et al. developed an intrinsic silicone shade guide tailored to the Indian demographic, featuring 15 distinct shades derived from a visual evaluation methodology. Their study revealed a range of agreement from minimal to moderate in color matching across a sample of 100 participants [8]. However, they did not fabricate swatches based on the suggested colors. Guttal et al. employed 10 powdered pigments to create silicone samples tailored for individuals with light, medium, and dark skin tones. A four-step wedge-wax pattern was fabricated with thicknesses of 1, 2, 4, and 6 mm. Color-matching assessments were conducted on three patients representing light, medium, and dark skin tones, and these evaluations were performed by four independent assessors. The inter-observer results for the four-step wedge silicone samples indicated moderate concordance for the light skin tone. For medium skin tone, kappa values demonstrated moderate to strong agreement. Evaluation of the silicone samples corresponding to dark skin tone revealed a high level of agreement [5]. Wee et al. proposed a shade guide by forming five clusters and five shades using a spectroradiometer [14]. Over et al. [15] employed a Minolta colorimeter to assess skin color in a cohort of 15 adult Caucasian subjects and developed a seven-step wedge silicone shade guide. Their findings indicated a strong correlation between the colorimeter assessments of the patients and the silicone samples, with the b* color dimension exhibiting the highest reproducibility, followed by the L* and a* dimensions. Silicone samples measuring 6, 8, and 10 mm demonstrated the most accurate match to the patients; thus, this investigation illustrated that silicone samples can be effectively replicated, provided an optimal patient-silicone correlation is established.

The range of skin colors analyzed in our study encompassed shades ranging from light brown to dark brown. This variability can be ascribed to the increased concentrations of eumelanin, a pigment that contributes to brown and black coloration, present in Indian skin tones [16]. The present study used color tabs of 4 mm thickness, similar to a study by Anitha et al. [8]. The reason for the near-perfect agreement noticed in our study could be due to the standardization of conditions for assessment such as lighting, observer training, and distance of the camera from the subject. Clinically, this high level of reliability ensures that assessments are consistent across observers, thus minimizing diagnostic errors or inconsistencies. This has direct implications for improving patient outcomes, particularly in fields where precise color assessments are integral.

Skin color is determined by a variety of factors including the type of melanin present, exposure to ultraviolet (UV) light, genetic makeup, the composition of melanosomes, and other pigments found in the skin. The perception of skin color is influenced in part by the presence of different combinations of four chromophores: carotenoids, melanin, oxyhemoglobin, and hemoglobin. This complex mixture of chromophores leads to variations in the way light is reflected and absorbed by the skin, resulting in a diverse range of skin tones. For instance, the combination of oxyhemoglobin and hemoglobin produces a pink hue in white skin, while the interplay between melanin and carotenes results in a yellow-orange tint in brown skin. Melanocytes, which are specialized skin cells responsible for producing the pigment melanin, play a crucial role in determining skin color. Within melanocytes, melanosomes are organelles that are involved in the synthesis, storage, and transfer of melanin pigment. Changes in skin color across different populations are primarily due to alterations in the size, distribution, and abundance of keratinocytes and melanocytes, rather than variations in the number of melanocytes present. In individuals with light skin tones, melanosomes are smaller and tend to be grouped together in secondary lysosomes, whereas those with darker skin tones have larger melanosomes that are dispersed individually within lysosomes [17].

Clinical implications of the study

The results of this investigation underscore the clinical significance of a tailored silicone shade guide specifically designed for Indian skin tones, facilitating accurate color matching for maxillofacial prosthetics. Its elevated inter-observer reliability reduces subjective discrepancies, thereby enhancing diagnostic precision, patient satisfaction, and therapeutic results in prosthetic rehabilitation, ultimately improving the quality and uniformity of care across various clinical environments.

Limitations of the study

This study has several constraints. First, the sample population may have inadequately captured the extensive variety of Indian skin tones, which could restrict the applicability of the customized shade guide. Second, the dependence on color tabs with a consistent thickness of 4 mm might overlook differences in translucency or dermal thickness across various anatomical areas. Moreover, the investigation did not evaluate the long-term stability or chromatic alterations in silicone materials resulting from environmental influences, such as UV radiation or the aging process. Finally, the assessment was performed under controlled conditions, which may not accurately reflect real-world clinical settings, where lighting and other factors can vary significantly.

## Conclusions

This investigation effectively established and substantiated a customized silicone shade guide comprising 24 shades specifically designed for the Indian demographic, classified into light, medium, and dark skin categories. Each category had eight subshades. The guide exhibited commendable inter-observer reliability and accuracy in color matching, underscoring its potential to enhance outcomes in maxillofacial prostheses. A high degree of agreement among observers regarding the satisfaction levels with the shade guide was noticed. The resilience and aesthetic versatility of silicone make it an exemplary material for precise shade replication. Subsequent research should aim to test the guide in a larger sample and augment the guide's relevance and longevity across various clinical environments.
